# Relation of Redox and Structural Alterations of Rat Skin in the Function of Chronological Aging

**DOI:** 10.1155/2019/2471312

**Published:** 2019-02-14

**Authors:** Aleksandra Jankovic, Luciano Saso, Aleksandra Korac, Bato Korac

**Affiliations:** ^1^Institute for Biological Research “Sinisa Stankovic”, University of Belgrade, Serbia; ^2^Department of Physiology and Pharmacology “Vittorio Erspamer”, Faculty of Pharmacy and Medicine, Sapienza University of Rome, Italy; ^3^Faculty of Biology, University of Belgrade, Serbia

## Abstract

Accumulation of oxidative insults on molecular and supramolecular levels could compromise renewal potency and architecture in the aging skin. To examine and compare morphological and ultrastructural changes with redox alterations during chronological skin aging, activities of antioxidant defense (AD) enzymes, superoxide dismutase (SOD), catalase (CAT), glutathione peroxidase (GSH-Px), glutathione reductase (GR), thioredoxin reductase (TR), and methionine sulfoxide reductase A (MsrA), and the markers of oxidative damage of biomolecules—4-hydroxynonenal (HNE) and 8-oxoguanine (8-oxoG)—were examined in the rat skin during life (from 3 days to 21 months). As compared to adult 3-month-old skin, higher activities of CAT, GSH-Px, and GR and a decline in expression of MsrA are found in 21-month-old skin. These changes correspond to degenerative changes at structural and ultrastructural levels in epidermal and dermal compartments, low proliferation capacity, and higher levels of HNE-modified protein aldehydes (particularly in basal lamina) and 8-oxoG positivity in nuclei and mitochondria in the sebaceous glands and root sheath. In 3-day-old skin, higher activities of AD enzymes (SOD, CAT, GR, and TR) and MsrA expression correspond to intensive postnatal development and proliferation. In contrast to 21-month-old skin, a high level of HNE in young skin is not accompanied by 8-oxoG positivity or any morphological disturbances. Observed results indicate that increased activity of AD enzymes in elderly rat skin represents the compensatory response to accumulated oxidative damage of DNA and proteins, accompanied by attenuated repair and proliferative capacity, but in young rats the redox changes are necessary and inherent with processes which occur during postnatal skin development. Мorphological and ultrastructurаl changes are in line with the redox profile in the skin of young and old rats.

## 1. Introduction

Many theories try to explain aging, at least the molecular basis of the pathophysiological processes (changes) that accompany it. One of them is the free radical theory postulated by Denham Harman [1], the universal theory that can be applied to all types of cells and tissues, particularly to the skin. The skin is one of the biggest organs in the body, accounting for 16% of body weight, protects against mechanical and radiation injuries and the entry of foreign substances into the body, serves as a sensory organ rich in nerves, regulates body temperature, and participates in the metabolism of fat (by forming depots) and in the metabolism of water and salt [2].

The skin is a mirror of aging [3]. Aging can be influenced by endogenous (intrinsic aging) and exogenous (extrinsic aging) factors. Oxidative pressure in skin which is exposed to high partial pressure of oxygen from the inside (circulatory system) and outside (50 times higher concentration) [4] can be increased by activation of molecular oxygen by absorption of light [5]. Other components in the skin (keratin proteins, hemoglobin, porphyrins, carotene, nucleic acids, melanin, lipoproteins, peptide bonds, and aromatic acids—tyrosine, tryptophan, histidine, and others) can also absorb light and transfer the excitation energy to molecular oxygen, creating singlet oxygen and other reactive species [6]. Also, the skin contains a high level of polyunsaturated fatty acids in membranes (25% of overall amount) and a high level of iron (it excretes around 0.24-0.6 mg daily) that can additionally increase oxidative pressure and reactive oxygen species (ROS) production [7].

It is unequivocally demonstrated that ROS are involved in many pathological conditions in the skin: immune system disorders [8], vitiligo [9], psoriasis [10], acne and ulcerations [11], rheumatoid arthritis [12], skin tumors [13], and others. Moreover, when affected by exogenous factors—drugs [14], UV radiation [15, 16], etc.—and endogenous factors—inflammation [17], ischemia/reperfusion [18], etc.—their production in the skin increases. In addition, in different animal models, morphological and structural alterations in the epidermis and dermis of aged skin are clearly shown.

However, the results of many studies examining the role of free radicals in the aging process are inconsistent, often contradictory. The inconsistency of the results is a consequence of the different *in vitro* or *in vivo* studies, examining different cell lines, tissues, and organs, parameters of oxidative damage and antioxidative defense (AD), as well as the period of the life cycle during which aging was examined.

In previous decades, only one paper has been published [19] which has comprehensively examined AD and markers of oxidative damage in animals as a function of chronological aging in female hairless mice aged 10 weeks (young) and 63 weeks (old). The authors conclude that neither the epidermis nor the dermis showed changes in activity/amount of key enzymatic and nonenzymatic components of AD, as well as in the lipid hydroperoxide levels between young and old mice (a histological comparison is absent in this study).

In the current study, chronological aging in rat skin was examined as a process inherent in life, in discrete time intervals since birth (3^rd^ day) to 21 months. In relation to the function of aging, we have examined (i) the activities of major antioxidant enzymes, superoxide dismutase (SOD), catalase (CAT), glutathione peroxidase (GSH-Px), glutathione reductase (GR), thioredoxin reductase (TR), and methionine sulfoxide reductase A (MsrA) and (ii) the markers of oxidative damage, 4-hydroxynonenal (HNE) and 8-oxoguanine (8-oxoG). We have tried to establish two additional imperatives in this study: firstly, to examine and compare morphological, structural, and ultrastructural changes with redox alterations during aging in skin and secondly, to take a look at and discriminate all examined parameters and processes during life (from 3 days to 21 months), comparing the changes in young (first two weeks of birth, postnatal period when the skin intensively develops) and old rats (rats aged 21 months) with those in adult animals (3 months).

## 2. Material and Methods

### 2.1. Animals and Sample Preparations

The experiments were approved by the Ethical Committee for the Treatment of Experimental Animals of the Institute for Biological Research “Siniša Stanković,” Belgrade. Healthy male Wistar rats were kept in a temperature-controlled room (24 ± 1°C) on a 12/12 reverse light/dark cycle, with standard chow diet and water ad libitum. Six individuals of the same age were housed per cage. The rats were killed on the same day by decapitation at different ages, from 3 days to 21 months.

The skin was dissected within 3 min of death and thoroughly rinsed with physiological saline to remove traces of blood. Parts of skin from the dorsal side were taken after cutting the fur with an electric clipper. The hypodermis was removed with a scalpel, and the remaining skin portions were prepared for enzyme activity analysis and light and electron microscopy.

For analysis of AD enzyme activities, a portion of the tissue weighing 1.5 g was homogenized (Ultra-Turrax homogeniser, Janke und Kunkel Ka/Werke, Staufen, Germany, 0-4°C) in 5 ml 0.25 M sucrose, 0.1 mM EDTA, and 50 mM Tris-HCl buffer (pH 7.4). The homogenates were sonicated and centrifuged as described previously [20]. Supernatants were used for determination of protein concentration and AD enzyme activities. The protein concentration in the supernatant was estimated by the method of Lowry et al. [21].

### 2.2. Activity of Antioxidative Defense Enzymes: SOD, CAT, GSH-Px, GR, and TR

Total specific SOD activity was determined by a modified method of Misra and Fridovich [22]. A degree of epinephrine autoxidation inhibition by SOD contained in the examined samples in 0.05 M sodium carbonate buffer (pH 10.2) was monitored spectrophotometrically at 480 nm. The unit of SOD was defined as the amount of enzyme that leads to 50% inhibition of the epinephrine autoxidation rate under the appropriate reaction conditions at 26°C. Catalase was assayed according to Beutler [23]. The method is based on the rate of H_2_O_2_ degradation by the action of catalase contained in the examined sample followed spectrophotometrically at 230 nm in 5 mM EDTA and Tris-HCl solution (pH 8.0). The catalase activity was calculated using a molar extinction coefficient for hydrogen peroxide (0.071), and values were expressed in units per mg of protein. One unit of catalase was defined as the amount of enzyme causing a decay of 1.0 *μ*mol of H_2_O_2_ per minute at 25°C, at a 10 mM hydrogen peroxide. To determine the activity of GSH-Px, a spectrophotometric method of Paglia and Valentine [24] was used. The method is based on the measurement of NADPH consumption (i.e., NADPH oxidation by GR (Sigma, type III product) at 340 nm). This reaction is preceded by the action of GSH-Px contained in the samples examined on *t*-butyl hydroperoxide (3 mM) as a substrate in 0.5 phosphate buffer (pH 7.0) at 37°C. The activity was expressed as nmol NADPH oxidized min^−1^ mg^−1^ protein. GR activity was assayed by the method of Glatzle et al. [25] and expressed as nmol NADPH min^−1^ mg^−1^ protein. This procedure is based on GR-catalyzed reduction of oxidized glutathione with NADPH, and oxidation of the latter was determined spectrophotometrically at 340 nm, using 2 mM GSSG and 0.1 mM NADPH, in phosphate buffer (pH 7.4), at 37°C. The method of Luthman and Holmgren [26] was used to determine TR activity. The method is based on the use of TR as a catalyst for the NADPH-dependent reduction of the disulfide bond in the 5,5′-dithio-bis-nitrosobenzoic acid (DTNB), with an increase in the absorbance at 412 nm over the first two minutes. The activity of the enzyme is determined by the amount of NADPH that is oxidized in a minute, since 1 mol of NADPH produces 2 mol of thienitrobenzoate and is expressed as a specific activity in nM NADPH min^−1^ mg^−1^ protein.

### 2.3. Morphological Analysis by Light and Electron Microscopy

Immediately after isolation, skin samples were fixed in 2.5% glutaraldehyde in 0.1 M Sørensen phosphate buffer (pH 7.2), postfixed in 2% osmium tetroxide in the same buffer, routinely dehydrated using increasing concentrations of ethanol, and embedded in Araldite (Sigma-Aldrich Laborchemikalien GmbH, Hamburg, Germany) as done previously [27]. Blocks were trimmed and cut into 1 *μ*m sections using a Leica UC6 ultramicrotome (Leica Microsystems, Wetzlar, Germany), mounted on copper grids, and contrasted in uranyl acetate and lead citrate using Leica EM STAIN (Leica Microsystems). Sections were examined on a Philips CM12 transmission electron microscope (Philips/FEI, Eindhoven, Netherlands) equipped with a digital camera (SIS MegaView III, Olympus Soft Imaging Solutions, Münster, Germany).

### 2.4. Immunohistochemistry

Semifine (1 *μ*m thick) sections were used for proliferating cell nuclear antigen (PCNA) and MsrA, HNE, and 8-oxoG detection by routine immunohistochemistry. After removal of Araldite with 1% sodium ethoxide in absolute ethanol (40 min, 37°C), skin sections were rehydrated and subjected to heat-mediated epitope retrieval and 3% hydrogen peroxide to block endogenous peroxidase staining. Subsequently, slides were immersed in protein block for 30 min at room temperature. After thorough rinsing, sections were incubated overnight at 4°C with anti-HNE (1 : 400; ab48506; Abcam, Cambridge, UK), anti-MsrA (1 *μ*g ml^−1^; ab16803, Abcam), anti-8-oxoG (1 : 100; ab65548, Abcam), or anti-PCNA (1 : 100; sc7907; Santa Cruz Biotechnology, Santa Cruz, CA, USA) antibodies. After rinsing in PBS, the sections were incubated with the avidin-biotin-peroxidase complex (ABC-peroxidase kit, Vector Labs, Burlingame, CA, USA), according to the manufacturer's instructions. After rinsing in PBS, the peroxidase reaction was developed using 3′-3′-diaminobenzidine tetrahydrochloride (Sigma-Aldrich, St. Louis, MO, USA) in the presence of hydrogen peroxide for 7 min in the dark. Negative controls were performed for each set of experiments by omitting primary antibodies and substituting them with PBS. All sections were counterstained with haematoxylin, dehydrated, and mounted for the analysis with an optical light microscope (Leica DMLB microscope, Leica Microsystems). The quantitative evaluation of antibody staining intensity in skin sections was analyzed as described previously [28] using the IHC Profiler plugin and open-resource digital image analysis software, ImageJ.

### 2.5. Propidium Iodide Staining

Semithin (1 *μ*m thick) sections were used for the analysis of chromatin condensation and apoptotic changes of nuclei. After removal of Araldite with 1% sodium ethoxide in absolute ethanol (40 min, 37°C), skin sections were rehydrated and stained with propidium iodide for 10 min. After rinsing in distilled water, the sections were mounted in Mowiol solution (Polysciences, Eppelheim, Germany) in order to preserve fluorescence signal and examined with a Carl Zeiss LSM 510 confocal laser scanning microscope (Carl Zeiss, Oberkochen, Germany).

### 2.6. Statistics

Statistical analyses were performed by one-way ANOVA followed by Dunnett's post hoc test using Prism version 5.00 software (GraphPad Software, USA). Measurements and quantifications are shown as mean ± SEM. Significance levels among different groups are indicated within the figure legends. An asterisk (∗) is used for indicating significance levels between an adult rat (3 m) and aging groups (3d-21 m). The *p* value < 0.05 was regarded as significant.

## 3. Results

### 3.1. Light and Electron Microscopy of Age-Related Structural Changes in the Rat Skin

**Figure 1:**
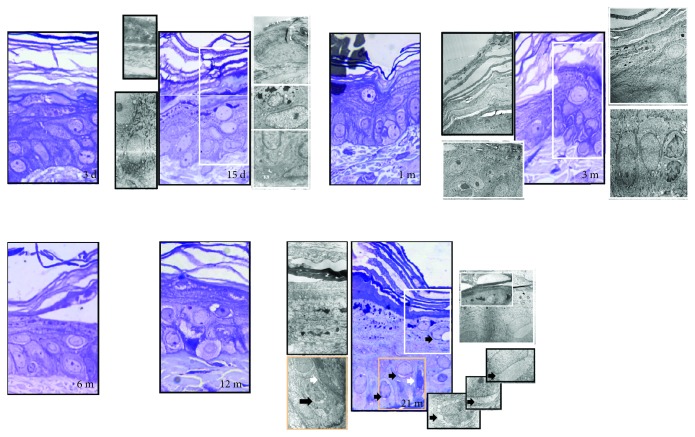
Light and electron microscopy of age-related structural changes in the rat skin. In the young skin, all epidermal cell layers have a normal structure and all cells appeared to be healthy (3 days, 15 days, 1 month, and 3 months old). Aging (6-month-, 12-month-, and 21-month-old skin) increases the epidermal layer thickness and the intercellular space throughout the epidermis along with a decrease in epithelial-dermal junction. In old skin, the basal lamina was partially detached and disrupted from the basal cell layer. Desquamation, through reduction in intercellular lipid contents, was seen in the upper layer of the stratum corneum. Intracytoplasmic vacuolization in the keratinocytes increased with aging. Namely, the keratinocyte mitochondria become light and swollen and concentrated around the nucleus to form an electron-lucent structure—“nuclear halo” (black arrow, 21 m, right insets). The numerous small cytoplasmic vacuoles, located near the nucleus, were observed in the basal keratinocytes gradually becoming a large cytoplasmic vacuole. Degenerative cells with a dark cytoplasm (white arrow, 21 m) were also seen in the basal layer of the epidermis. Semifine sections stained with toluidine blue, magnification: ×100, orig.; transmission electron microscopy, magnification: ×8000, orig.

**Figure 2:**
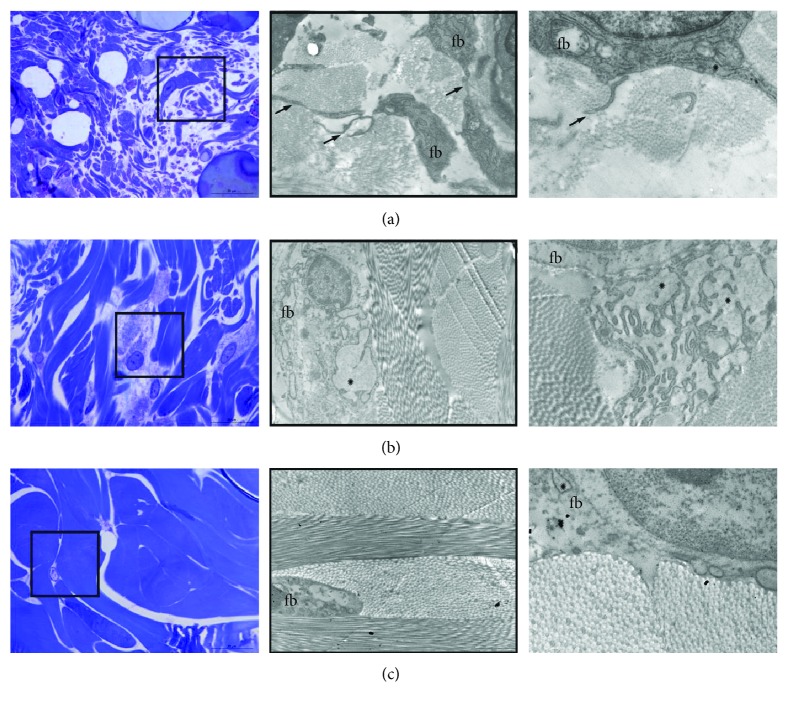
Lifetime ultrastructural characteristics of fibroblasts and collagen. The dermis in the skin of 15-day-old rats is occupied with numerous elongated fibroblasts (a). The extracellular matrix is poorly organized, and sparse thin collagen bundles are visualized between fine fibroblast extensions (arrows). The mature skin dermis in adult 3-month-old rat (b) consisted of large fibroblasts with well-developed rough endoplasmic reticulum (ER). Dilated ER cisterns are filled with proteinaceous material; the extracellular matrix is well organized, showing compact and regularly oriented collagen bundles (b). In the dermis of 21-month-old rat, the fibroblasts reduced in number and their long cytoplasmic extensions wrap large collagen bundles with thicker and tightly packed, but poorly oriented and electron-lucent, fibers (c). Magnification: semifine section, toluidine blue staining: ×100, orig.; framed area analyzed on transmission electron microscopy, magnification: ×3000, orig.; far right: ×8800, orig.

The thickening of the epidermis, with morphological alterations of the stratum basale, the disappearance of interkeratinocytes and dermoepidermal junctions, empty perinuclear spots in keratinocytes, and the disappearance of the basal lamina were evident with advancing age (Figures 1 and 2). Besides, the dermal matrix was more disorganized and avascular with the reduced number of fibroblasts in old, as compared to mature (adult) and young skin (Figures 1 and 2). The mitochondrial destruction was evident in keratinocytes, fibroblasts, and mastocytes (Figures 1 and 2).

### 3.2. Ultrastructural Characteristics of Fibroblasts and Collagen during Lifetime

Numerous elongated fibroblasts fulfill the dermis of 15-day-old rats (Figure 2(a)). The extracellular matrix is poorly organized, and sparse thin collagen bundles are visualized between fine fibroblast extensions (arrows in Figure 2(a)). The dermis in an adult 3-month-old rat consisted of large fibroblasts with well-developed rough endoplasmic reticulum (ER). Dilated ER cisterns are filled with proteinaceous material; the extracellular matrix is well organized, showing compact and regularly oriented collagen bundles (Figure 2(b)). However, in the dermis of a 21-month-old rat, fibroblasts reduced in number and their long cytoplasmic extensions wrap large collagen bundles with thicker and tightly packed, but poorly oriented and electron-lucent, fibers (Figure 2(c)).

### 3.3. Proliferative Capacity of Skin Cells during Lifetime

**Figure 3:**
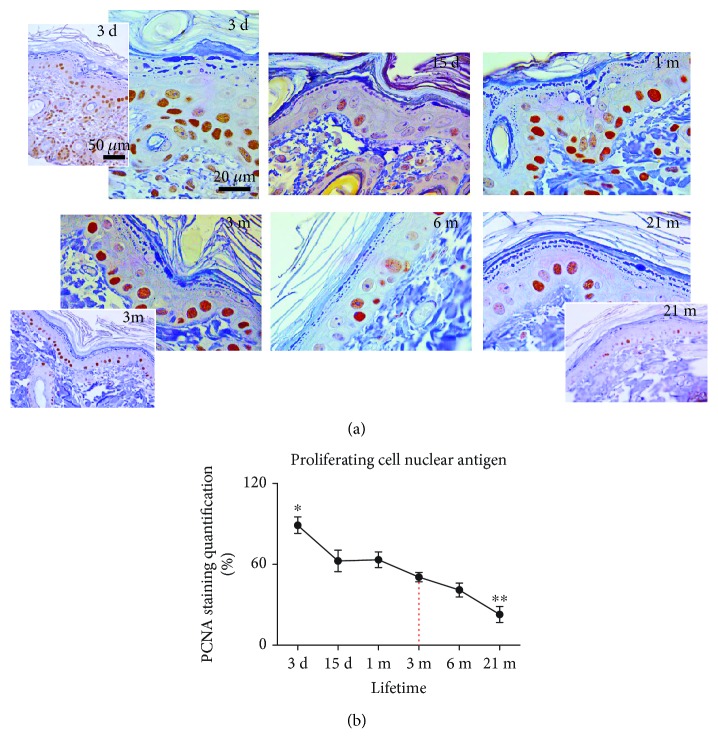
Expression of proliferating cell nuclear antigen (PCNA) in the skin of 3-day-, 15-day-, 1-month-, 3-month-, 6-month-, and 21-month-old rats. Tissues were subjected to immunohistochemistry using a PCNA-specific antibody. The brown nuclei testify to the specific staining (a). The quantitative evaluation of PCNA antibody staining intensity in skin sections was analyzed by the IHC Profiler using the open-source ImageJ program. The relative mean intensity was determined from 6 images for each aging group (*n* = 6), and it is shown graphically (b). Data were presented as mean ± SEM. ^∗^In respect to 3-month-old rats; ^∗^*p* < 0.05; ^∗∗^*p* < 0.01. ×100 magnification, orig.; corner insets show low magnification (×40, orig.) of representative skin sections.

Immunohistochemical analysis revealed the highest expression of PCNA in the skin of postnatal 3-day-old rats (Figure 3). At this age, a plethora of PCNA-immunopositive nuclei were evident in the epidermis and dermis (Figure 3(a), inset). The expression of PCNA was detected in nuclei of basal keratinocytes, scattered dermal fibroblasts, and cells of the dermal papilla in adult (3-month-old) rats. The PCNA immunopositivity was evidently diminished in the epidermis and dermis of elderly (21-month-old) rat skin (*p* < 0.01).

### 3.4. Time-Course Changes in the Level of HNE-Modified Proteins in the Rat Skin

**Figure 4:**
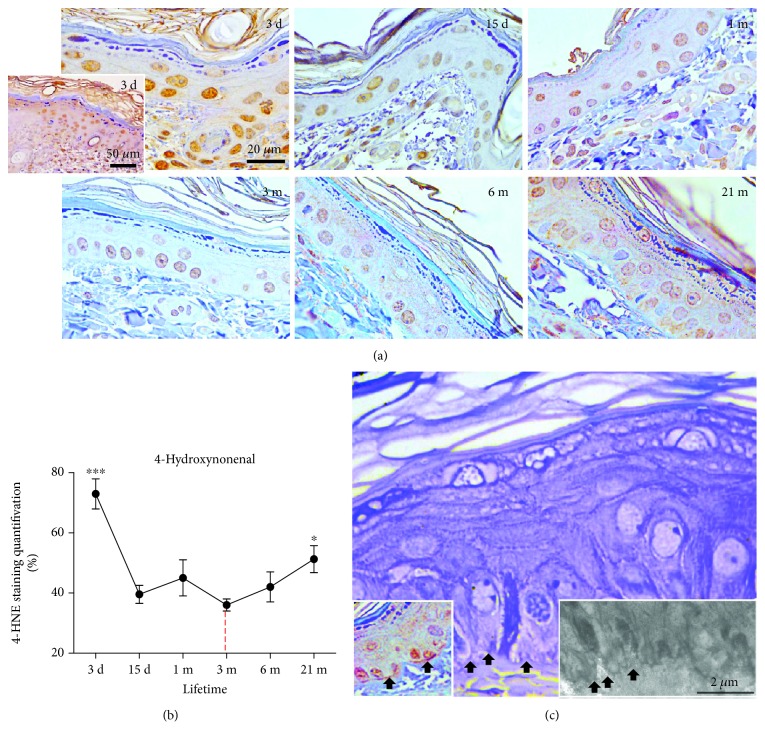
Time-course changes in the levels of HNE-modified proteins in the skin of 3-day-, 15-day-, 1-month-, 3-month-, 6-month-, and 21-month-old rats. The immunohistochemical method was used to detect skin tissue proteins modified by 4-hydroxynonenal (HNE) for subsequent quantitative image analysis (b). The brown staining testifies to the specific reaction. Strong HNE immunopositivity of the basal lamina, along with its partial discontinuation/disruption (black arrows), was observed in 21-month-old rat skin (c, left inset, arrows). Dermoepidermal junctions and the basal cell deep projections into the dermis were also disturbed (c, middle and right, arrows). (a) Representative slides of HNE immunohistochemistry are shown at ×40 (corner inset) and ×100 magnification, orig. The quantitative evaluation of HNE antibody staining intensity in skin sections (b) was analyzed as described in Figure 3. (c) Magnification: semifine section, toluidine blue staining: ×100, orig. Insets: left—HNE immunohistochemistry: ×100 orig.; right—transmission electron microscopy, bar: 2 *μ*m. The relative mean intensity was determined from 6 images for each aging group (*n* = 6). Data were presented as mean ± SEM. ^∗^In respect to 3-month-old rats; ^∗^*p* < 0.05; ^∗∗∗^*p* < 0.001.

The highest level of HNE-protein adducts was observed in postnatal 3-day-old rats (Figure 4(b)). This level was higher compared to that in young adult 3-month-old rat (*p* < 0.001) and all other ages (Figures 4(a) and 4(b)). In 3-day-old skin, strong HNE immunopositivity was observed both in the cytoplasm and in the nuclei of epidermal and dermal cells (Figure 4(a)). In adult (3-month-old) rat skin, only nuclei in the stratum basale were faintly HNE-positive. In comparison to young adult skin, an increased granular, mostly cytoplasmic reaction for HNE was evident in 21-month-old rat skin (*p* < 0.05). Intensive HNE immunoreactivity in the basal lamina was also apparent in some areas of the 21-month-old skin (Figure 4(c), right inset).

### 3.5. Analysis of the DNA Lesion and 8-OxoG in the Skin of Rats during the Lifetime

**Figure 5:**
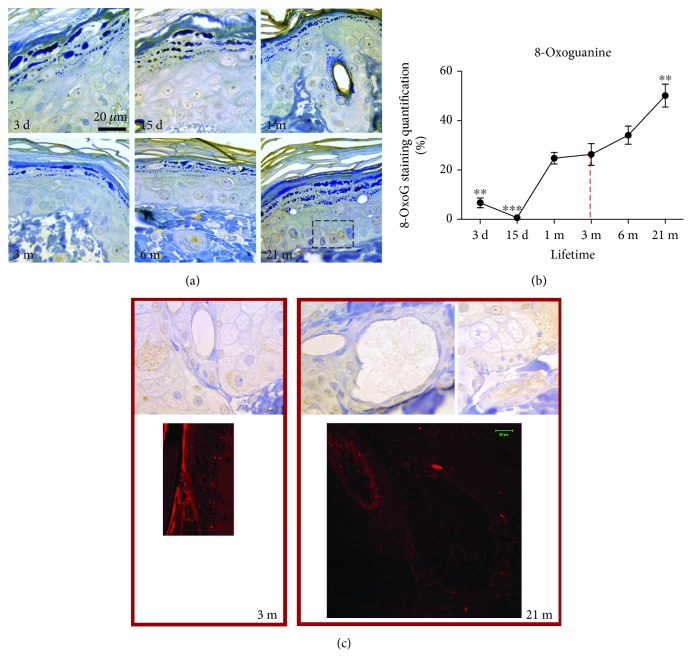
Analysis of the DNA lesion and 8-oxoguanine (8-OxoG) in the skin of rats during the lifetime. Tissues were subjected to immunohistochemistry using an 8-OxoG-specific antibody. The brown staining testifies to the specific reaction (a). The quantitative evaluation of 8-OxoG antibody staining intensity in skin sections was analyzed as described in Figure 3 (b). The relative mean intensity was determined from 6 images for each aging group (*n* = 6). Data were presented as mean ± SEM. ^∗^In respect to 3-month-old rats; ^∗∗^*p* < 0.01; ^∗∗∗^*p* < 0.001. The mitochondrial positivity of 8-OxoG corresponds to apoptotic positivity observed by propidium iodide staining in the sebaceous glands and root sheath (c, right). Magnification: ×100, orig.

Aging increases the number of 8-OxoG-immunopositive nuclei in epidermal cells (from 1 m to 21 m), but only in the skin of 21-month-old rats is immunopositivity of 8-OxoG present in all epidermal layers, including the stratum basale (Figure 5(a)). In comparison to young adult skin, immunopositivity of 8-OxoG was higher in 21-month-old rat skin (*p* < 0.01) (Figure 5(b)). Besides, the mitochondrial positivity of 8-OxoG is observed in 21-month-old skin, and this positivity corresponds to apoptotic positivity observed by propidium iodide staining in the sebaceous glands and root sheath (Figure 5(c)).

### 3.6. Age-Dependent Changes of the SOD, CAT, GSH-Px, GR, and TR Activities in the Skin of Rats

**Figure 6:**
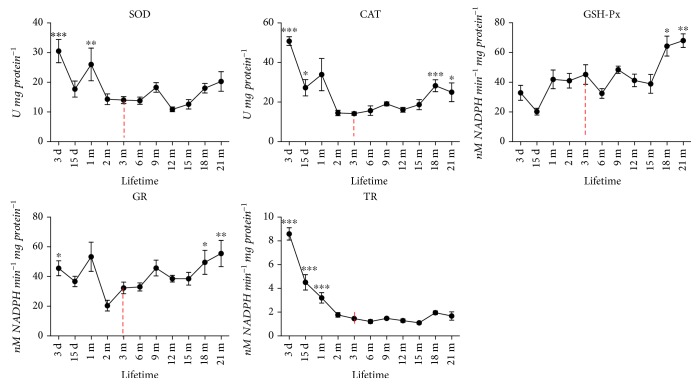
Age-dependent changes of the total superoxide dismutase (SOD), catalase (CAT), glutathione peroxidase (GSH-Px), glutathione reductase (GR), and thioredoxin reductase (TR) activities in the skin of rats. Data were presented as mean ± SEM. ^∗^In respect to 3-month-old rats; ^∗^*p* < 0.05; ^∗∗^*p* < 0.01; ^∗∗∗^*p* < 0.001.

In comparison to adult (3-month-old) skin, total SOD and TR activities were higher in the preadult period: SOD activity was higher in 3-day- and 1-month-old groups (*p* < 0.001 and *p* < 0.01), while TR was significantly higher in 3-day-, 15-day- and 1-month-old groups (*p* < 0.001) (Figure 6). Thereafter, activities of SOD and TR remain constant through adulthood and old age. Activities of CAT and GR decrease sharply towards adulthood (2-3 m) and mostly remain constant in middle-aged rat skin but thereafter increase again in the oldest groups (18-month- and 21-month-old groups). In a similar fashion, activity of GSH-Px was higher in the oldest groups of rats, as compared to the young adult group (3 months old).

### 3.7. Immunoexpression of MsrA

**Figure 7:**
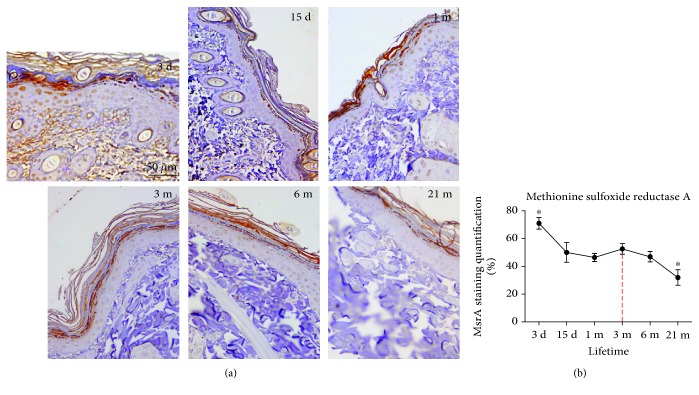
Expression of methionine sulfoxide reductase A (MsrA) in the skin of 3-day-, 15-day-, 1-month-, 3-month-, 6-month-, and 21-month-old rats. Tissues were subjected to immunohistochemistry using an MsrA-specific antibody. The brown staining testifies to the specific reaction. The quantitative evaluation of MsrA antibody staining intensity in skin sections (b) was analyzed as described in Figure 3. The relative mean intensity was determined from 6 images for each aging group (*n* = 6). Data were presented as mean ± SEM.^∗^In respect to 3-month-old rats; ^∗^*p* < 0.05. Representative slides are shown at ×40 magnification, orig.

The highest level of MsrA is detected in 3-day-old (early postnatal) skin (Figure 7(a)). Strong immunostaining for MsrA is evident in both epidermal and dermal skin compartments. The MsrA expression decreases with passage of life, and the lowest level of MsrA is observed in the skin of old 21-month-old rats (Figure 7(b)).

## 4. Discussion

Aging studies were mostly based on comparison of AD and lipid peroxidation in only two age groups of animals [19] or fibroblasts from young and old donors [29]. In this study, the relation of redox and structural alterations in skin aging was examined in light of chronological aging, in discrete time intervals since birth (3^rd^ day) to 21 months of rat age. In comparison to adult (3-month-old) skin, higher activities of enzymes involved in ROS removal are observed in young (3-day-old) and old (21-month-old) skin. In old skin, increases in CAT, GSH-Px, and GR are associated with a decline in protein expression of MsrA, accumulation of HNE-modified protein aldehydes, particularly in the basal lamina, and 8-oxoG positivity in nuclei and mitochondria. Such redox profile in old skin is accompanied by degenerative changes at structural and ultrastructural levels in epidermal and dermal compartments (thickening of the epidermis, occurrence of inter- and intracellular empty spaces throughout the epidermis, disappearance of the basal lamina, loss of dermoepidermal junction, rarefaction of fibroblasts, and enlargement and thickening of collagen bundles) and low renewal skin capacity (according to a decrease in the number of PCNA-positive cells). In 3-day-old skin, higher SOD, CAT, GR, and TR activities and MsrA protein expression correspond to intensive postnatal development and proliferation. Moreover, the high level of HNE characterizes young skin, but it is not accompanied by any signs of oxidative insults on DNA, cell, or tissue structure levels. The overall results of this study indicate that “positive” oxidative pressure (or eustress) seems to be integral in adaptive physiology of a newborn's skin. Also, progressively compromised redox flexibility and/or repair, rather than an overall drop in ROS scavenging capacity and/or severe oxidative injuries, reflect acceleration of chronological aging of the skin in adults. The details of the redox states in the skin and their correlations with morphofunctional changes in the function of lifetime are discussed in more detail below.

Histology of old animals shows typical aging changes in the structural organization of the skin, and all of them can result from the action of free radicals. Damage to the extracellular matrix by free radicals, especially hyaluronic acid and proteoglycans [30], can result in the thickening of the epidermis and the empty spaces between keratinocytes, as noted in old skin (Figure 1). Within the keratinocytes, mitochondria become light, swollen, and concentrated around the nucleus resulting in the occurrence of degenerative changes, known as perinuclear “halo” formations (Figure 1). This histological phenomenon also occurs in UV-irradiated skin, and it closely correlates with ROS levels, since pretreatment with SOD restricts the formation of “halo” spaces [31]. The loss of normal maturation and irregularities in the epidermal layers, as noted here in the skin of old rats, is common in photodamaged skin and is also connected to free radical action [15, 32]. In the dermis of old rats, a decline in collagen fiber content and density is pronounced (Figure 2). It is well known that increased ROS, as in inflammatory conditions, can affect collagen density directly by damaging fibers and indirectly by interfering with collagen synthesis [33] or fibroblast proliferation and increasing senescence [34, 35].

The senescent phenotype is characterized by low expression of proteins required for DNA synthesis and low proliferation rate [36]. In agreement, the lower number of PCNA-positive nuclei in the dermis and epidermis is found in 21-month-old rat skin (Figure 3). Accumulation of molecular oxidative damage (oxidative modifications of membrane lipids, DNA, and proteins) accelerates replicative senescence of skin fibroblasts *in vitro* and aging *in vivo* [37–39]. In turn, senescent fibroblasts are a source of ROS, as reviewed in [40]. Thus, a vicious cycle between ROS-induced senescence and increased ROS due to accumulation of senescent cells could be established in aged skin.

In parallel with morphofunctional manifestations of detrimental ROS action, we examined the level of HNE-protein adducts. HNE, produced during the lipid peroxidation process, covalently binds to proteins to form Michael adducts [41]. An increase in HNE-modified proteins is common upon serial passaging of human keratinocytes [42] or replicative senescence of WI-38 fibroblasts [35] and in the retina of aged rats [43]. Thus, HNE is an important marker and propagator of lipid, DNA, and protein oxidation [44–46] and is of particular importance in aging skin. Results of this study show that in comparison to adult skin, HNE immunopositivity increases in old age (after 21 months). The observed increase in HNE immunopositivity in old skin could however be “diluted,” since skin consists of many layers and structures, and modifications of some of them may severely inflict the whole skin architecture. In line with this, strong HNE immunopositivity detected on the basal lamina, uneven in some parts of skin, may contribute to its deterioration with advanced aging (as shown in Figure 4(c)).

An increase in HNE-modified proteins with aging could be regarded as a consequence of diminished antioxidant protection. However, no significant changes were found in the case of SOD and TR, and the activities of CAT, GSH-Px, and GR even show an increase in old (18 m-21 m) rat skin (Figure 6). Although studies reported species-, strain-, sex-, and tissue-specific age-related changes in AD [47], the skin of old mice [19] and fibroblasts from old human donors [29] are mostly characterized by unchanged or increased AD enzyme activities. Moreover, fibroblasts from old donors are more resistant to oxidative pressure from hydrogen peroxide, linoleic acid hydroperoxide, or UV light, presumably because of higher activity of GSH-Px in these cells [29]. It remains vague however whether the increase in HNE, observed in the current study in old skin, is associated with imperfect ROS scavenging or rather it is predominantly determined by impaired degradation of HNE-modified proteins in aging skin, as suggested [42], or by both. In addition, concomitant with accumulation of oxidative damages on molecular and supramolecular levels, the skin from 21-month-old rats is characterized by low expression of MsrA. This enzyme is essential in the maintenance of functional proteins in aerobic cells [48, 49] since it catalyzes Trx-dependent reduction of methionine sulfoxide to methionine [50, 51]. Thus, a decline in MsrA expression in old rats, as observed here in the skin of old rats (Figure 7) and other aging tissues or senescent cells [52, 53], suggests that a decline in protein repair contributes to the accumulation of oxidized proteins and impaired redox sensitivity/regulation with chronological skin aging, regardless of overall upregulation of AD enzyme activities. Moreover, a progressive, generalized impairment of oxidative repair could result in an increase in 8-oxoG positivity in nuclei of basal keratinocytes and mitochondria in the secretory cells of sebaceous glands observed in the 21-month-old skin (Figure 5).

To better understand the process of chronological skin aging, the current time-course study puts forward the dynamics of changes in the skin redox state and its correlation with morphofunctional characteristics of the skin in discrete time intervals during life, including the changes immediately after birth.

According to a plethora of PCNA-positive nuclei, the earliest postnatal development is characterized by the intensive proliferation rate of skin cells. Intensive proliferation enables thickening of the outer layer (stratum corneum) and dermis (growth of hair follicles, sebaceous and sweat glands) as presented in Figures 1–3. These processes are essential in the development of skin barrier function—defense of the organism against low/high temperature, oxygen, and UV radiation after birth. It seems that this adaptive period in rats lasts for a few weeks, since PCNA immunopositivity progressively diminished starting from 15 days on (Figure 3). A similar time frame exists in mice where the maximal proliferation of dermal fibroblasts is seen in newborns, but it decreased as early as the third postnatal week, the same time when the dermis reaches its maximal thickness [54].

In parallel, marked HNE immunopositivity in early postnatal skin (3 days old), as seen in Figure 4, could reflect the intensive metabolic activity needed for ongoing growth, proliferation, and morphogenesis of epidermal layers and dermal structures (like hair follicles and glands). Also, higher HNE may be connected to the increase in ROS production imposed by exposure of the skin to a new environment, oxygen, and UV light, following the transition from intrauterine to extrauterine environment. These external factors seem to be important sources of lipid peroxides and HNE in skin [55]. A “physiologically relevant increase” in HNE levels, triggered by a rise in partial oxygen pressure, could support proliferation/cell turnover in postnatal skin. Accordingly, higher levels of HNE and AD in postnatal skin, as compared to adult skin, are not surprising. The role of HNE in proliferation and differentiation of cells *in vitro* is well recognized but complex, which is dependent on multitude variables, including the cellular level of HNE, redox environment, and cell type [56–58]. Thus, additional studies are needed to comprehend physiological signaling of HNE in postnatal skin.

In summary, the results of this study indicate that chronological aging of the skin is associated with slowly advancing redox “inflexibility” and repair, rather than with an overall drop in ROS-scavenging capacity. Although similar internal and external sources may increase ROS in young and senescent skin, the mechanisms for their removal are different, and so is the result accordingly: proliferation/regeneration in postnatal skin and senescence and atrophy in the elderly. Understanding the regulation of redox-recycling-redox systems, like those with reductase and denitrosilase activity (Msr, GSH/GR, and thioredoxin/thioredoxin reductase) and genome-repairing systems (such as 8-oxoguanine DNA glycosylase), can advance healthy aging of the skin.

## 5. Conclusion and Perspectives

Without any intention to overestimate the importance of the free radical theory of aging over others, it is possible to suggest that observed morphological and ultrastructural changes in old skin are a consequence of elevated oxidative pressure and that the relation between the increased markers of oxidative damage and the following compensatory elevation of AD cannot be excluded. A similar conclusion, but in the other direction, is valid when it comes to young skin. Even though the free radical theory of aging is limited in providing us with all of the answers regarding aging, our study shows that altered redox equilibrium in skin has a role in the development of this process throughout life, despite some assumptions that Harman's theory is overcome or dead [59]. Perhaps, the best question would be, How long would we live with reduced or no AD? Possibly, just like erythroid progenitor cells without manganese SOD, we ended up dying before maturation [60]. In any case, research should be focused on this direction, when aging and free radical theory of aging are in question.

## Data Availability

The data used to support the findings of this study are included within the article.
